# Supplementary material for: The impact of regular school closure on seasonal influenza epidemics: a data-driven spatial transmission model for Belgium
Source: BMC Infect Dis. 2018 Jan 10;18:29. doi: 10.1186/s12879-017-2934-3 (PMC5764028; doi:10.1186/s12879-017-2934-3)
Supplement: Additional file 1 — The Additional File provides additional details on the modeling approach, the calibration procedure, as well as additional validation results. (PDF 839 kb) [file 12879_2017_2934_MOESM1_ESM.pdf]

ADDITIONAL FILE
The impact of regular school closure on seasonal influenza epidemics: a data-driven spatial transmission model for Belgium

Giancarlo De Luca[1], Kim Van Kerckhove[2], Pietro Coletti[2], Chiara Poletto[1], Nathalie Bossuyt[3], Niel Hens[2,4], and Vittoria Colizza[1,5]

[1]Sorbonne Universités, UPMC Univ Paris 06, INSERM, Institut Pierre Louis d'Epidémiologie et de Santé Publique (IPLESP, UMR−S 1136), Paris, France
[2]Interuniversity Institute for Biostatistics and statistical Bioinformatics, Hasselt University, Belgium
[3]Scientific Institute of Public Health (WIV-ISP), Public health and surveillance Directorate, Epidemiology of infectious diseases Service, Brussels, Belgium
[4]Centre for Health Economics Research and Modelling Infectious Diseases, Vaccine and Infectious Disease Institute, University of Antwerp, Belgium
[5]ISI Foundation, Turin, Italy

1 Metapopulation model

1.1 Commuting by age

We describe here in detail the inference procedure used to approximate fluxes of commuters per age class $i = a, c$ in Belgium. For each commuting link l of the French commuting network, we computed the commuting distance $d(l)$ and the fraction $\rho(l)$ of commuters of age class i. We filtered all links having less than 30 commuters. We considered seven bins of distance, according to the definition used in the "Enquête National Transport et Déplacements 2008" (National survey on transport and mobility, 2008): $[0, 2]$ km, $]2, 5]$ km, $]5, 10]$ km, $]10, 20]$ km, $]20, 40]$ km, $]40, 80]$ km, and > 80 km. The distribution obtained for each distance bin is then used to infer the fraction of Belgian commuters in age class i traveling on the same distance bin. A comparison between the empirical distributions obtained from the French commuting data and the reconstructed distributions for Belgium is shown in Figure S1. A good agreement is found for all distance bins, with a noisier behavior obtained for the bin class $d(l) \leq 2$km, due to poor statistics. Plots also show that children commute at shorter distances than adults.

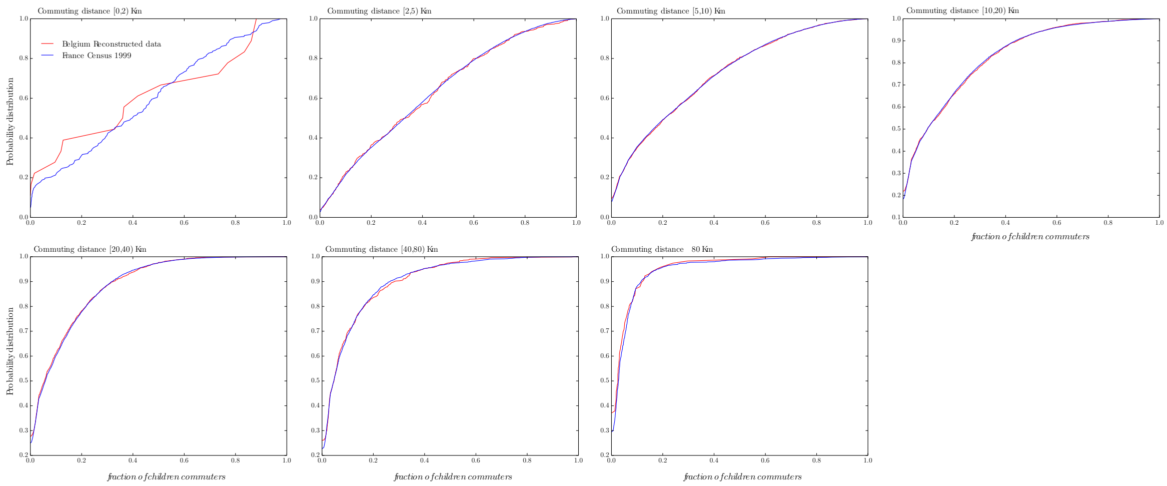

Figure S1: Probability distribution of the fraction of children commuters at specific distance bins: comparison between the empirical distributions obtained from the French commuting data and the reconstructed distributions for Belgium.

1.2 Contact Matrices

The average number of contacts made by participants in age class $i = 1, 2$ with people in age class $j = 1, 2$ is given by M_{ij}. The per capita contact rates are then summarised in the contact rate matrix

$$\bar{C}_{ij} = M_{ij}/N_j,$$

which is rescaled to a normalised contact matrix

$$C_{ij} = \bar{C}_{ij} \cdot N_{\text{tot}},$$

where N_{tot} is the total population of Belgium

We note here that the values C_{ij} are scale invariant, that is

$$C_{ij} = \frac{M_{ij}}{N_j} N_{tot} = \frac{M_{ij}^{(p)}}{N_j^{(p)}} N_{tot}^{(p)}. \tag{S1}$$

We consider C_{ij} to describe the social interaction in each patch, i.e. $C_{ij}^{(p)} = C_{ij}$, so that $\bar{C}_{ij}^{(p)} = \frac{C_{ij}}{N^{(p)}(t)}$ following Eq.(S1), with $N^{(p)}(t)$ being the total population in the patch at time t.

1.3 Details of the compartmental model in each patch

Each patch receives commuters from $k_{in}(p)$ patches and moves residents to $k_{out}(p)$ other patches, with k_{in} and k_{out} representing the indegree and outdegree, respectively, of patch p in the commuting network. Commuters are modeled with separate compartments, in order to track them in their movements from residence to destination and back. At each time step t, the population of a patch p is composed of the following subpopulations, each described by a two-age class SEIR disease progression model:

- individuals who reside in patch p and do not commute: $S_i^{p|p\to p}$,$E_i^{p|p\to p}$,$I_i^{p|p\to p}$,$R_i^{p|p\to p}$, for each age class $i = c, a$;
- $k_{out}(p)$ subpopulations of individuals who reside in patch p and commute to another patch q: $S_i^{p|p\to q}$, $E_i^{p|p\to q}$, $I_i^{p|p\to q}$, $R_i^{p|p\to q}$, with q neighbor of p, and $i = c, a$;
- $k_{in}(p)$ subpopulations of individuals who reside in a patch q and commute from patch q to patch p: $S_i^{p|q\to p}$, $E_i^{p|q\to p}$, $I_i^{p|q\to p}$, $R_i^{p|q\to p}$, with q neighbor of p, and $i = c, a$.

Accounting for commuting, we can then write the force of infection for a susceptible individual of age class i in patch p and time t:

$$\lambda(i,p,t) = \beta \sum_j C_{ij}(t) \frac{I_j^p(t)}{N^p(t)}, \tag{S2}$$

with

$$I_j^p(t) = I_j^{p|p\to p} + \sum_q I_j^{p|p\to q} + \sum_q I_j^{p|q\to p}\,. \tag{S3}$$

1.4 Influenza transmission

Here we describe influenza transmission for the subpopulation of residents of a given patch p (we drop the p for simplicity). The extension to the other subpopulations present in the patch is straightforward. The probabilities associated to SEIR transitions for age class i in a small enough time interval dt are given by:

- $p_i^{S\to E}(t) = 1 - e^{-\beta\lambda(i,t)dt}$;
- $p_i^{E\to I}(t) = 1 - e^{-\epsilon dt}$;
- $p_i^{I\to S}(t) = 1 - e^{-\mu dt}$.

The number of individuals in age class i newly entering the E, I, and R class are extracted with binomial distributions (B):

- $E_{new,i} \sim B(p_i^{S\to E}(t), S_i^{p|p\to p}(t))$;
- $I_{new,i} \sim B(p_i^{E\to I}(t), E_i^{p|p\to p}(t))$;
- $R_{new,i} \sim B(p_i^{I\to R}(t), I_i^{p|p\to p}(t))$;

1.5 Derivation of the next generation matrix

In calculating the values of R we disregard mobility. The in-patch model becomes therefore a two age-classes stochastic SEIR model. Its deterministic couterpart can be written as:

$$\dot{\vec{S}}(t) = -\beta\vec{S}\frac{\bar{C}(t)}{N^p}\vec{I} \tag{S4}$$

$$\dot{\vec{E}}(t) = \beta\vec{S}(t)\frac{\bar{C}(t)}{N^p}\vec{I}(t) + \epsilon\vec{E}(t) \tag{S5}$$

$$\dot{\vec{I}}(t) = \epsilon\vec{E}(t) - \mu\vec{I}(t) \tag{S6}$$

$$\dot{\vec{R}}(t) = \mu\vec{I}(t) \tag{S7}$$

where $\vec{S} = \begin{pmatrix} S_c \\ S_a \end{pmatrix}$, $\vec{E} = \begin{pmatrix} E_c \\ E_a \end{pmatrix}$, $\vec{I} = \begin{pmatrix} I_c \\ I_a \end{pmatrix}$, and $\vec{R} = \begin{pmatrix} R_c \\ R_a \end{pmatrix}$.

Using Diekmann's approach we linearize the equations of the infectious compartments $\vec{E}, \vec{I}$ around the disease free state with the correct immunity fraction $\vec{S}_o = \begin{pmatrix} (1-g_c)N_c^p \\ (1-g_a)N_a^p \end{pmatrix}$,$\vec{E}_0 = \vec{I}_0 = \begin{pmatrix} 0 \\ 0 \end{pmatrix}$ and $\vec{R}_0 = \begin{pmatrix} g_c N_c^p \\ g_a N_a^p \end{pmatrix}$ and obtain for the following system of linear equations restricted to the infectious compartments:

$$\dot{\vec{E}}(t) = \beta \begin{pmatrix} (1-g_c)N_c^p & 0 \\ 0 & (1-g_a)N_a^p \end{pmatrix} \frac{C(t)}{N^p} \vec{I}(t) + \epsilon \vec{E}(t) \tag{S8}$$

$$\dot{\vec{I}}(t) = \epsilon \vec{E}(t) - \mu \vec{I}(t). \tag{S9}$$

The next generations matrix in the patch therefore reads ($\boldsymbol{I}$ is the identity matrix):

$$\begin{aligned} \boldsymbol{K}^p &= \frac{\beta}{N^p} \begin{pmatrix} (1-g_c)N_c^p & 0 \\ 0 & (1-g_a)N_a^p \end{pmatrix} \boldsymbol{C(t)} \cdot (\epsilon \boldsymbol{I})^{-1} \cdot (\epsilon \boldsymbol{I}) \cdot (\mu \boldsymbol{I})^{-1} = \\ &= \frac{\beta}{\mu} \begin{pmatrix} (1-g_c)\frac{N_c^p}{N^p} & 0 \\ 0 & (1-g_a)\frac{N_a^p}{N^p} \end{pmatrix} \boldsymbol{C(t)} \end{aligned} \tag{S10}$$

which in components gives the result reported in the main text:

$$K_{ij}^p = \frac{\beta}{\mu}(1-g_i)\frac{N_i^p}{N^p} C_{ij}(t) \tag{S11}$$

1.6 Computational details

The code of the simulations was written in C++ and made use of the Mersenne-trwister random generator and the binomial extraction procedures as provided by the Boost Libraries v1.58.0. Compiling was done using the gnu c++ compiler version 4.8.1 with optimization level 3.

1.7 List of districts

Table S1 presents the list of district names and associated IDs used in the study.

2 Calibration procedure

We minimized the Weighted Least Square function $WLS(\beta_n, \alpha_n)$ computed on the median normalized incidence curves, considered from the start of the epidemic up to the peak time. The calibration is performed on Brussels district only and for each set of parameters (β_n, α_n) we performed 1,000 simulations. Here β_n is the explored per-contact transmission rate, and α_n is the rescaling factor for the simulated incidence in Brussels district to account for possible sampling biases in the initial condition. The calibration is performed on normalized incidence curves to discount the effects of unknown GP consultation rates.

To reduce the number of points to explore and cope with stochastic fluctuations we considered iterative resampling through a particle filter/bootstrap method. For each level l we calculate a weight distribution for each (β_n, α_n) as follows:

$$w_l(\beta_n, \alpha_n) = \frac{\frac{1}{WLS(\beta_n,\alpha_n)}}{\sum_{\beta_n,\alpha_n} \frac{1}{WLS(\beta_n,\alpha_n)}}, \tag{S12}$$

which allows us to define a filtering/resampling transition probability:

$$p(\beta, \alpha; l+1 \mid \beta_1^l, \dots, \beta_n^l, \alpha_1^l, \dots, \alpha_n^l; l) = \sum_{\beta_n^l, \alpha_n^l} w_l(\beta_n^l, \alpha_n^l) V_{\beta_n,\alpha_n)}(\beta, \alpha) \tag{S13}$$

where $V_{(a_0,b_0)}(a, b)$ is the uniform distribution over the Voronoi cell centred in (a_0, b_0). We can then resample N-particles at level $l+1$ given the M-particles at level l and repeat the process iteratively until the filtering

ID	District	alternative name	ID	District	alternative name
0	Antwerpen	Anvers	22	Charleroi	
1	Mechelen	Malines	23	Mons	Bergen
2	Turnhout		24	Moeskroen	Mouscron
3	Brussel	Bruxelles	25	Soignies	Zinnik
4	Halle-Vilvoorde	Hal-Vilvorde	26	Thuin	
5	Leuven	Louvain	27	Tournai	Doornik
6	Nivelles	Nijvel	28	Huy	Hoei
7	Brugge	Bruges	29	Liège	Luik
8	Diksmuide	Dixmude	30	Verviers	
9	Ieper	Ypres	31	Waremme	Borgworm
10	Kortrijk	Courtrai	32	Hasselt	
11	Oostende	Ostende	33	Maaseik	
12	Roeselare	Roulers	34	Tongeren	Tongres
13	Tielt		35	Arlon	Aarlen
14	Veurne	Furnes	36	Bastogne	Bastenaken
15	Aalst	Alost	37	Marche-en-Famenne	
16	Dendermonde	Termonde	38	Neufchâteau	
17	Eeklo		39	Virton	
18	Gent	Gand	40	Dinant	
19	Oudenaarde	Audenarde	41	Namur	Namen
20	Sint-Niklaas	Saint-Nicolas	42	Philippeville	
21	Ath	Aat			

Table S1: List of district names and associated IDs.

probability is almost uniform, and therefore the filter does not work any more. Here we used 20 particles at each level (except the first) and then we stopped when the number of effective particles defined as:

$$N_{\text{eff}} = \frac{1}{\sum_{\beta_n^l} w_l(\beta_n^l, \alpha_n^l)^2} \tag{S14}$$

was greater of 19, which corresponds to uniformity of filtering probability. For each level l, we thus obtain a set of 20 pairs (β_n^l, α_n^l) whose distribution is used to estimate the values of α and β that minimize $WLS(\beta, \alpha)$.

3 Additional validation results

Calibration results are listed in Table S2. Figure S2 shows the comparison in the peak timing between simulations calibrated with values of Table S2 and surveillance data.

Table S2: Calibration results.

	median	95% CI
β	0.0850	$0.0842, 0.0860$
α	0.068	0.061–0.073
WLS	0.0068	0.0063–0.0115

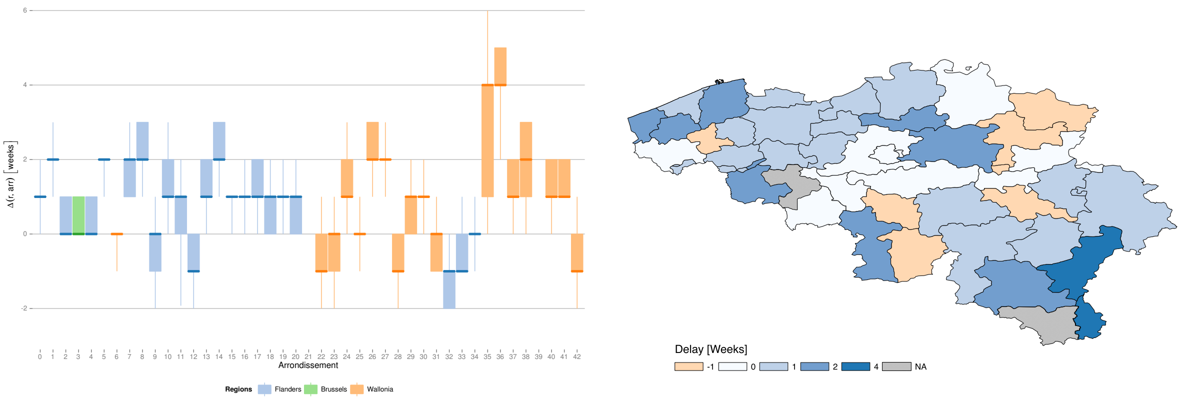

Figure S2: Left: Boxplot of the peak time difference ΔT^d per district between simulations and empirical data. Numbers represent Belgium districts, see Table S1 for corresponding names. Right: Geographical map of the median peak time difference per district.
